# Metabolite-driven remodeling of hepatic lipid metabolism by the plasticizer di-isononyl phthalate

**DOI:** 10.1016/j.molmet.2026.102412

**Published:** 2026-07-03

**Authors:** Sini Pitkänen, Henriikka Hakomäki, Olli Kärkkäinen, Marko Lehtonen, Sreejita Das, Jaana Rysä, Jenni Küblbeck, Anna-Liisa Levonen

**Affiliations:** 1 A. I. Virtanen -institute for Molecular Sciences, University of Eastern Finland, Kuopio, Finland; 2 School of Pharmacy, University of Eastern Finland, Kuopio, Finland

**Keywords:** Di-isononyl phthalate, Liver, Lipid metabolism, Mitochondria, Metabolism-disrupting chemical

## Abstract

**Introduction:**

Phthalates are widely used as plasticizers in consumer products and are suspected to be metabolism-disrupting chemicals. Di-isononyl phthalate (DINP) is commonly recognized as less hazardous substitute for more studied di(2-ethylhexyl) phthalate (DEHP).

**Materials and methods:**

The effects of DINP on hepatic lipid metabolism were studied using C57BL/6J mice with diet-induced obesity, and human HepaRG and C3A cell lines. The mice were orally exposed to 0, 1.5, 15 or 150 mg/kg bw/d DINP for 20 weeks, followed by assessment of glucose and insulin tolerance, hepatic histology, transcriptome and metabolome. The cells were exposed to DINP and its metabolites, followed by measurement of mitochondrial function and nuclear receptor activation.

**Results:**

The highest dose of DINP decreased hepatic lipid droplets and slightly attenuated weight gain and glucose tolerance of the mice. DINP exposure elevated acylcarnitine levels, indicating altered fatty acid beta-oxidation, which was accompanied by enrichment in mitochondrial and peroxisomal lipid metabolism pathways at transcriptomics level. *In vitro*, monoisononyl phthalate (MINP), the primary metabolite of DINP, increased mitochondrial respiration and beta-oxidation in presence of long-chain fatty acids. DINP metabolites activated peroxisome proliferator-activated receptors (PPARs) of both mouse and human, with an activation profile partially distinct from DEHP.

**Conclusions:**

Our findings indicate that DINP remodels hepatic lipid metabolism through its active metabolites via PPARs at high doses, with additional modes of action at lower exposure levels. Due to species-specific differences in nuclear receptor activation potencies, the adverse or potentially beneficial nature of these effects in humans remains ambiguous.

## Highlights

• Common plasticizer DINP induced lipid beta-oxidation and affected hepatic lipid deposition in obese mice
• Acylcarnitine levels were increased in the liver and plasma after DINP exposure
• DINP metabolites activated mouse and human PPARs with an activation profile partially distinct from DEHP

## Graphical abstract

**Figure:**
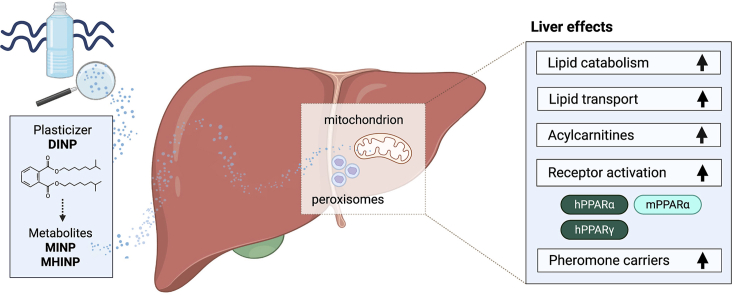

## 1 Introduction

Metabolic diseases such as metabolic dysfunction-associated steatotic liver disease (MASLD), hyperlipidemia, obesity and type II diabetes (T2D) present significant global health challenges affecting millions of people worldwide [1,2]. MASLD is the most common chronic liver disease with estimated 38 % global prevalence in adults [3]. It is a progressive disease which may develop into metabolic dysfunction-associated steatohepatitis (MASH) or even cirrhosis and hepatocellular carcinoma [4]. The hallmark of MASLD is lipid accumulation in the liver tissue due to increased lipid uptake and biosynthesis next to impaired lipid metabolism and transport outside of the liver [5]. MASLD is considered to be a hepatic manifestation of metabolic syndrome, as it often co-occurs with other metabolic comorbidities such as hyperglycemia, insulin resistance, dyslipidemia and obesity [4].

Phthalates are synthetic plasticizers and dissolving agents which are commonly used in manufacturing of common consumer products, such as plastics and cosmetics [6]. They are suspected of being so-called metabolism-disrupting chemicals (MDCs), characterized by the ability to interfere with metabolic functions and increase the risk for developing metabolic disorders [7]. Exposures to commonly used phthalates, such as di(2-ethylhexyl) phthalate (DEHP), have been shown to disturb lipid and glucose metabolism in multiple cell and animal studies [[8], [9], [10]]. Importantly, phthalate exposure has been associated with MASLD in several epidemiological studies [11]. Similar links have been found with insulin resistance and metabolic syndrome [12,13].

Di-isononyl phthalate (DINP) is a high molecular weight phthalate, which has been used as substitute for DEHP. DINP is lesser studied compared to DEHP, but these molecules share similarities in their physicochemical properties. Metabolism of phthalates is typically rapid – in the first phase, the parental diester phthalate undergoes hydrolysis in the gastrointestinal tract, and upon the release of the alkyl group, it can be absorbed as the corresponding monoester phthalate, which is mono(2-ethylhexyl) phthalate (MEHP) for DEHP and monoisononyl phthalate (MINP) for DINP [14]. These primary metabolites undergo further metabolic reactions in the liver, which leads to production of several potentially active metabolites.

Mouse models of diet-induced obesity (DIO) have been shown to be useful for the studies inspecting chemically induced metabolic adverse effects [15,16]. The metabolic challenge with high-fat diet (HFD) shifts the energetic balance and may sensitize the mice to further chemically induced metabolic disruption. Furthermore, HFD mimics the modern dietary status which is characterized by high intake of fat, sugar and processed food [17]. In the DIO models, various metabolic effects have been demonstrated after chronic DEHP exposures. For example, in C57BL/6J female mice, chronic DEHP exposure reduced the metabolic rate, which accelerated weight gain and lipid accumulation, and led to impaired glucose tolerance [18]. In contrast, C57BL/6J male mice demonstrated reduction in plasma triglycerides, body weight and total body fat after DEHP exposure [19]. In another study with C57BL/6J males, DEHP exposure disrupted hepatic lipid metabolism by promoting both lipogenesis and fatty acid oxidation at the same time, and the effect was dependent on dietary status, as it was only detected in obese mice [20]. To our knowledge, DINP exposure studies have not been previously conducted using DIO models.

To fill the gap in knowledge about DINP metabolic effects, we conducted a 20-week exposure study using 1.5, 15 and 150 mg/kg bw/d doses of DINP and inspected glucose and insulin tolerance, histology, transcriptome and metabolome, focusing on the liver tissue. We then compared the responses to human hepatic cell lines, by assessing the effects of DINP and its metabolites on mitochondrial function, the rate of beta-oxidation and species-specific nuclear receptor activation. Overall, this study demonstrated the capacity of DINP metabolites to remodel hepatic lipid metabolism and mitochondrial function in mouse liver and human hepatic cells, highlighting several modes of action at different exposure levels.

## 2 Materials and methods

### 2.1 Animals

60 wildtype C57BL/6J male mice were acquired from The Jackson Laboratory (France). All procedures involving animals were conducted under EU legislation and approved by the National Animal Experiment Board of Finland, under licenses ESAVI/42100/2022 and ESAVI/43804/2019.

### 2.2 Cell culture

Undifferentiated human hepatic HepaRG cells (BPHPR101) at p.12 were acquired from Biopredic International (Saint Grégoire, France) and human hepatoma C3A cells (CRL-10741) from ATCC/LGC Standards (UK). HepaRG cells were cultured in William’s E medium (Gibco 22551-022) supplemented with 10 % FBS (Gibco 10500-064), 1 % penicillin-streptomycin (Gibco 15120-122), 1 % L-glutamine (Gibco 25030081), 1 % human hydrocortisone (Cayman Chemicals 18226) and 0.05 % human insulin (Gibco 12585-014), and C3A cells in DMEM (Gibco 11880) supplemented by 10 % fetal bovine serum (FBS, Biowest S181B, Nuaillé, France), 1 % L-glutamine (Biowest X0550), and 1 % penicillin/streptomycin (Biowest L0022), in a humidified atmosphere at 37 °C with 5 % CO_2_. HepaRG cells were differentiated in batches using a standard protocol, allowing them to proliferate for 14 days in the growth media, followed by a 14-day differentiation by 1.7 % DMSO (Sigma Aldrich D8418) [21]. Differentiated HepaRG cells were used in experiments in passage 18, and C3A cells between passages 7 to 14.

### 2.3 Test chemicals

Technical-grade DINP (P0300, mixture of branched chain isomers) was obtained from TCI Chemicals (Japan), MINP (1,2-benzenedicarboxylic acid 1-(7-methyloctyl) ester, TRC-M547540), monohydroxyisononyl phthalate (MHINP, TRC-M547540) and MEHP (Ehrenstorfer 16178900) from LGC Standards, phthalic acid (402915), DEHP (Supelco 67261), 1,2-cyclohexane dicarboxylic acid diisononyl ester (DINCH, Y0002022) and fenofibrate (F620) from Sigma Aldrich, and PPARα inhibitor GW6471 (HY-15372), PPARγ inhibitor GW9662 (HY-16578) and bezafibrate (HY-B0637) from MedChemExpress. Test compound stocks and dilutions were prepared in DMSO (Sigma Aldrich D8418).

### 2.4 *In vivo* study design

6-week-old mice were transported and acclimated to the local lab animal center habitat for two weeks before starting the experiment. The mice were single housed in individually ventilated polysulfone cages (Tecniplast 1285) with *ad libitum* access to tap water from metal-capped drinking bottles. The mice were maintained in a room with controlled temperature (22 ± 2 °C), humidity (55 ± 15 % RH) and a 12/12h light-dark cycle. From the age of 8 weeks, mice were fed *ad libitum* high-fat diet (HFD) with 45 % kcals from fat (Research Diets D12451). The general condition of the mice was inspected daily, and the bodyweights were recorded weekly. Average food intake was measured by recording the difference between food provided and remaining at each diet refresh, which was performed three times a week. The diet was distinguishable from the bedding material by its pink color.

The mice were habituated to daily consumption of palatable placebo pellets for 10 days before starting DINP exposure via these pellets (section 2.5.). At the end of the habituation period, the mice were randomized into three dose groups and a placebo control group (n = 14-15) using RandoMice [22]. Out of the initial 60, three mice were excluded from the experiment due to injuries due to transport-related stress (n = 1), significantly higher bodyweight (n = 1), and abnormal liver structure (n = 1). For 20 weeks, the mice were orally exposed to DINP every morning at 8–10 AM by voluntary ingestion of the DINP-containing pellet. Daily administration of the pellet was visually confirmed.

Intraperitoneal glucose and insulin tolerance tests (i.p.GTT and i.p.ITT) were conducted after exposure weeks 17 and 19. After 20 weeks of exposure, the mice were euthanized by CO_2_ inhalation. The whole blood was drawn by cardiac puncture, followed by PBS perfusion. The tissues were weighted and either flash frozen in liquid nitrogen for gene expression, untargeted metabolomics and biochemical analyses or immersed in fixative for histology.

### 2.5 *In vivo* DINP exposure

Gelatin pellets were prepared by mixing warm blackcurrant juice with gelatin (1.2 g gelatin in 10 ml of 50 % juice) and coagulating the mixture in glass-coated 96-well plates with U-shaped bottom (Thermo Fisher Scientific) [23]. These pellets were used as a vehicle to administer three doses of DINP corresponding to 1.5, 15 or 150 mg/kg bw/d. The control group received a placebo pellet. The pellets were prepared in batches and stored on glass dishes at −20 °C until administration. The middle DINP dose reflects the no-observed adverse effect level (NOAEL) established by EFSA (2019) and EPA (2022) based on chronic hepatic and renal effects in rats in a 2-year chronic study [24]. The highest DINP dose represented the mechanistic dose and provided a reference for more human-relevant lower doses. Typical phthalate exposure level is at μg/kg level in humans, but may rise for example in occupational setting, or in other less typical exposure scenarios, such as during hospitalization through i.v. medical devices [25].

### 2.6 Intraperitoneal glucose and insulin tolerance tests

The mice were fasted for 4–5 h before i.p.GTT and i.p.ITT. Around 30 min before the start, they were given local anesthetics (EMLA cream with 25/25 mg/g lidocaine/prilocaine, Aspen Pharma) on the tail tip. The baseline glucose was measured from small tail bleeding which was incised using a sterile scalpel. At 0 min, the mice were injected i.p. with 1.0 g/kg of 20 % glucose in 0.9 % NaCl in i.p.GTT or 0.5 mIU/g of 100 mIU/ml insulin (Actrapid Penfill 100 IU/ml, Novo Nordisk) in 0.9 % NaCl in i.p.ITT. Plasma glucose levels were measured from the tail bleeding at 15, 30, 45, 60, 90, and 120 min in i.p.GTT and at 15, 30, 45, 60, and 90 min in i.p.ITT.

### 2.7 Histology

#### 2.7.1 Paraffin-embedded sections

Collected tissues from the liver left lobe were fixed for 24 h at 4 °C in 4 % paraformaldehyde, processed and embedded in paraffin blocks. Sections of 5 μM were mounted on charged microscopy slides and stained with Delafield’s hematoxylin & eosin (H&E), Periodic acid-Schiff (PAS), and Masson’s trichrome (MT), using Trichrome Stain (Masson) Kit (Sigma–Aldrich).

#### 2.7.2 Cryosections

Collected tissues were flash frozen in liquid nitrogen and stored in −80 °C, embedded in Optimal cutting temperature (OCT) compound and sectioned in 8 μM thickness using cryotome with an ambient temperature of −22 °C. To stain neutral lipids in the liver (medial lobe), sections were fixed with formal calcium, stained with Oil-Red-O (ORO) and counterstained with Mayer’s hematoxylin.

#### 2.7.3 Imaging and analysis

The stained sections were imaged using VS200 slide scanner (Olympus) and quantitatively analyzed using OlyVIA (Olympus) and ImageJ/Fiji [26] softwares. Macro- and microvesicular steatosis were also visually scored using tiered grading system: 0 = not present (<5 %), 1 = mild (5–33 %), 2 = moderate (33–66 %) and 3 = severe (>66 %), and inflammation on a scale of 0 = not present, 1 = mild (<2 foci per field), 2 = moderate (2–4 foci per field) and 3 = severe (>4 foci per field) [27].

### 2.8 Quantitative triglyceride measurement

Triglycerides (TGs) were quantified from plasma and liver medial lobe homogenate using Liquicolor Mono (Human GmbH) kit, following manufacturer’s instructions, as described before [23]. Absorbance values of the liver were normalized to total protein content of the tissue, which was quantified using Pierce BCA Protein Assay Kit (Thermo Scientific 23225).

### 2.9 Determination of ALT in mouse plasma

Alanine transaminase (ALT) enzyme level in mouse plasma was measured using Mouse ALT (Alanine Transaminase) ELISA Kit (FineTest EM0829) by following manufacturer’s instructions.

### 2.10 Untargeted UPLC-MS/MS analysis

Cold acetonitrile was added into the plasma at a ratio of 200 μL per 50 μL of plasma. The samples were mixed by pipetting and then centrifuged for 10 min at 13 000 rpm at 4 °C. They were then stored at 10 °C until analysis. Frozen liver right lobes were weighed (134–402 mg wet weight), placed in methanol (80 %) at a standard concentration of 100 mg/300 μl solution, and then homogenized with Bead Ruptor 24 Elite (Omni International, Kennesaw, GA, USA). Tissues were homogenized in microtubes (2 ml, reinforced with 1.4 mm ceramic beads, Omni International, Kennesaw, GA, USA) with a cooling (2 °C) and a program dedicated to liver tissue (1 cycle, 30 s, speed 5). After homogenization, samples were transferred to shaker for 5 min (Multi Reax, Heidolph, Schwabach, Germany). Then samples were centrifuged for 10 min (14000 rpm at 4 °C) (Centrifuge 5804R, Eppendorf, Hamburg, Germany). The homogenized sample was diluted with methanol (80 %, 1:1, v/v) and transferred into filter plates (Captiva ND filter plate 0.2 μm) and mixed by pipetting. The samples were then centrifuged for 5 min at 700×*g* at 4 °C and kept at 10 °C until analysis.

A small portion of each sample was combined and utilized as a quality control (QC) sample. These QC samples were incorporated in the analysis at the beginning to equilibrate the analytical platform and subsequently after every 9 samples. Samples were randomized before the analysis. Plasma and liver samples had independent quality control samples.

Instrumental analysis was performed at the LC-MS metabolomics center (Biocenter Kuopio, University of Eastern Finland, Finland). The analysis was carried out using an ultra-high performance liquid chromatography (LC) system (Vanquish Flex UHPLC, Thermo Scientific, Bremen, Germany), which was coupled online to a high-resolution mass spectrometry (MS, Q Exactive Classic, Thermo Scientific). The samples were measured using two distinct chromatographic techniques: reversed phase (RP) and hydrophilic interaction chromatography (HILIC). The analyses in both chromatographic techniques include the utilization of both electrospray ionization (ESI) polarities, which were ESI positive (ESI+) and ESI negative (ESI-). The centroid mode was used to obtain full scan data. The data was collected over a mass to charge ratio (*m*/*z*) range of 120–1200 in the RP technique and 50 to 700 in the HILIC technique. To identify metabolites, we acquired data-dependent product ion spectra (MS/MS) from pooled QC samples at the beginning and end of the analysis for each mode. The configuration and specifications for the LC-MS instrument have been previously published [28].

The data was acquired in RAW file format and transformed into mzML using MSConvertGUI (ProteoWizard) [29]. Peak picking and alignment were done using MS-DIAL software (v.4.9.221218). The minimum peak height was set at 200 000 in positive mode and 100 000 in negative mode. With both modes, the minimum peak width was 8, retention time tolerance 0.5 min and peak count filter 50 %. The gaps were filled by compulsion. The peak alignment and metabolite annotation was done using a combination of in-house libraries and data from public databases. The alignment result was exported as peak areas for preprocessing and statistical analysis, in which the notame R package was used [30]. In preprocessing, the analytical modes were merged, and the data was corrected for analytical drift. The features with low detection rate (RSD rate <0.5 and D ratio <0.8) were flagged. Missing values were imputed using random forest imputation. The statistical analysis was done using Welch’s one-way ANOVA with Benjamini-Hochberg correction for false discovery rate (FDR). Metabolite identification was focused on features with statistically significant differences between the DINP-exposed groups and the control group. In the groupwise comparison, the features with FDR <0.05 were considered significant, and features with p < 0.05 and FDR >0.05 were considered as trends. Metabolites were identified based on the standard metabolite identification levels, in which 1 = metabolites confidently identified against in-house library, 2 = putatively annotated metabolites and 3 = putatively characterized metabolite classes [31]. The untargeted metabolomics data has been deposited at the NIH Common Fund's National Metabolomics Data Repository (NMDR) website, the Metabolomics Workbench [32], where it has been assigned to Project ID ST004436.

### 2.11 RNA sequencing

Total RNA was isolated from homogenized liver medial lobe using NucleoSpin RNA kit (Macherey Nagel). RNA sequencing libraries were prepared from the total RNA using QuantSeq 3′ mRNA-Seq V2 Library Prep Kit FWD with UDI (Lexogen), following the manufacturer’s instructions. The libraries were sequenced at Novogene (Munich, Germany) using Illumina’s PEI150 sequencing strategy in NovaSeq X plus sequencer. The reads were preprocessed using nf-core/rnaseq pipeline v. 3.14.0 [33], using STAR, RSEM and Salmon packages combined with quality control, and annotated to the GRCm38 reference genome. Differential gene expression analysis between the DINP-exposed groups and the control group was performed using DESeq2 [34]. Gene set enrichment analysis (GSEA) [35] was used to identify enriched pathways in the DINP-exposed groups. The pathways with FDR <0.05 were considered significantly altered. The data was visualized using ggplot2 [36] and GraphPad Prism v. 10.0.2. The data has been deposited at Gene Expression Omnibus (GEO) database under accession number GSE313258.

### 2.12 RT-qPCR

C3A cells were seeded on 12-well plates and transfected with silencer RNA for PPARA (Ambion AM16708; ID 142804) or negative control (Ambion 4390847), using Lipofectamine RNAiMAX (Thermo Fisher Scientific) for 48 h following the manufacturer’s recommendations. Cells were then incubated with test chemicals for additional 24 h, followed by RNA extraction by High Pure RNA Isolation Kit (Roche). RNA from C3A cells and mouse liver (Section 2.10.) was reverse-transcribed to cDNA by Transcriptor First Strand cDNA Synthesis Kit (Roche). Gene expression was assessed using Universal Probe Library (Roche) for *PLIN2* or PrimeTime Std qPCR Assays (Integrated DNA Technologies) for other genes on QuantStudio 3 system (Applied Biosystems). Primer sequences are described in Table 1. Gene expression was normalized to *PPIA/Ppia* (human/mouse) and quantified using the 2−ΔΔ*Ct* method.

**Table 1:** Sequences of RT-qPCR primers.

Gene	Forward primer	Reverse primer
*Mouse Ppia*	5′-CAAACACAAACGGTTCCCAG-3′	5′-TTCACCTTCCCAAAGACCAC-3′
*Mouse Ehhadh*	5′-GTGGAGCAAATGACAACTTCTG-3′	5′-TGGCTTCTGGTATCGCTGTA-3′
*Mouse Cyp4a10*	5′-TTCTGGGGAAGCAAGGC-3′	5′-TCCATTCAACAAGAGCAAACC-3′
*Mouse Cyp3a11*	5′-AGTAGCACACTTTCCTTCACC-3′	5′-CCATCTCCATCACAGTATCATACG-3′
*Human PPIA*	5′-CATCCTAAAGCATACGGGTCC-3′	5′-TCTTTCACTTTGCCAAACACC-3′
*Human ACOT1*	5′-TATTTGGTCTGGCTGATCGG-3′	5′-TCTGCCCAACTCTTCCTCT-3′
*Human ACOT2*	5′-TAGGCACAGAACATACTTGAGG-3′	5′-CTTCAGGCTCCATTGGTACAG-3′
*Human PLIN2*	5′-TCAGCTCCATTCTACTGTTCACC-3′	5′-CCTGAATTTTCTGATTGGCACT-3′

### 2.13 Western Blot

After siRNA transfections (section 2.12.), protein levels of PPARα in C3A cells were assessed using Western Blot. The cells were collected in RIPA buffer (20 mM Tris–HCl, 150 mM NaCl, 1 mM EGTA, 1.0 % NP-40, 1 % sodium deoxycholate, 2.5 mM sodium pyrophosphate, 1 mM b-glycerophosphate, 1 mM Na_3_VO_4_, 1 μg/ml leupeptin) with 1 mM PMSF and phosphatase inhibitor cocktail (Roche). The samples were sonicated for 5 min in 30 s on/off cycles and centrifuged at 14 000 rpm at 4 °C for 30 min to separate the supernatant, from which the protein levels were measured using Pierce BCA Protein Assay Kit (Thermo Fisher Scientific). Equal amounts of protein (20 μg) were suspended in Laemmli sample buffer, and the samples were denatured at 95 °C for 5 min. The proteins were separated by 10 % SDS-PAGE and transferred to nitrocellulose membranes (BioRad 1620112). Primary antibodies for PPARα (Invitrogen PA1-822A, 1:500 in 1 % BSA-TBST; a kind gift from Pasi Tavi, University of Eastern Finland) and beta-actin (ACTB, Invitrogen MA1-91399, 1:5000 in 1 % BSA-TBST) were incubated overnight, followed by TBST washes and a 1-hour incubation with HRP-conjugated secondary antibodies (Cell Signaling 7074S, 1:10 000 in 1 % BSA-TBST and R&D Systems HAF007, 1:10 000 in 1 % BSA-TBST). The blots were imaged using Western Lightning Ultra Chemiluminescent Substrate (NEL112001EA, Revvity) in ChemiDoc MP Imaging System (BioRad). Band intensities of PPARα were quantified using ImageJ/Fiji and normalized to ACTB. The silencing efficiencies at RNA and protein levels (51 and 41 % at 72 h) are shown in Figure S1.

### 2.14 Mitochondrial function assays

Differentiated HepaRG cells were seeded on XF96 cell culture microplates (Agilent 101085-004) to a density of 27 500 cells per well, in line with the smaller well area on the Seahorse plate reflecting 40 % of the suggested cell number for HepaRG in normal 96-well plate. The growth media was refreshed the next day and two days after that. On day 4, the cells were adapted to treatment media including William’s E (Gibco 22551-022) supplemented with 2 % FBS (Gibco 10500-064), 1% penicillin-streptomycin (Biowest L0022), 1 % L-glutamine (Gibco 25030081), 1 % human hydrocortisone (Cayman Chemicals 18226), 0.05 % human insulin (Gibco 12585-014) and 0.5 % DMSO (Sigma Aldrich D8418). On day 5, the cells were exposed to phthalates and PPAR inhibitors for 72 h. The final DMSO concentration in the media was constantly 0.5 % during the exposure.

Oxygen consumption rate (OCR) and extracellular acidification rate (ECAR) were measured after 72-hour exposure using the Seahorse XFe96 analyzer (Agilent). Before the assay, the treatment media was aspirated, the cells were gently washed with XF medium including DMEM with pH 7.4 (Agilent 103575-100) supplemented with 10 mM glucose (Gibco A24940-01), 1 mM pyruvate (Gibco 11360-039) and 2 mM glutamine (Gibco 25030081) in Mito Stress Test, or 2 mM glutamine (Gibco 25030081), 0.5 mM l-carnitine (Sigma Aldrich C0283) and 200 μM BSA-conjugated palmitate (Cayman Chemicals 29558) in beta-oxidation test. Fresh XF medium with phthalates was then added to the wells, and the cells were incubated in CO_2_-free atmosphere for 45 min. During the Mito Stress Test, respiration-modulatory compounds oligomycin A (Sigma Aldrich 75351) at 1 μM, FCCP (Sigma Aldrich C2920) at 2 μM, and antimycin A (Sigma Aldrich A8674) combined with rotenone (Sigma Aldrich R8875) at 0.5/0.5 μM final concentrations were sequentially injected to measure basal respiration, spare respiratory capacity, ATP production and coupling efficiency. During the beta-oxidation assay, mitochondrial beta-oxidation inhibitor etomoxir (Cayman Chemicals 11969) at 4 μM and antimycin A combined with rotenone at 0.5/0.5 μM final concentrations were sequentially injected to measure basal respiration, OCR corresponding to mitochondrial beta-oxidation, and non-mitochondrial oxygen consumption. Beta-oxidation was measured as the difference between OCR before and after the etomoxir injection (between time points 5 and 11). OCR was normalized to fluorescence signal from Hoechst 33342 (Invitrogen H3570).

### 2.15 Nuclear receptor activation assay

Nuclear receptor (NR) activation was assessed using luminescence-based reporter system in C3A cells seeded onto 96-well plates (62500 cells/cm^2^) in culture medium (1.2) 20–24h before transfection (other NRs) or modified culture medium containing 5 % of charcoal-stripped FBS (CF-FBS, Biowest S181F) (glucocorticoid receptor, GR). The cells were transfected for 4 h with 100 ng pCMVβ (Clontech Inc, [37]) and NR expression vectors and luciferase reporters (Table 2), using Lipofectamine 3000 (Invitrogen L3000, Thermo Fisher Scientific) with a ratio of 1 μl lipofectamine and 2 μl P3000 for 1 μg of total plasmid DNA. DMSO stocks of test chemicals and positive control agonists were diluted 1:1000 in DMEM supplemented with 5 % of charcoal-stripped FBS. After 24-hour exposure, the cells were lysed with and frozen at −80 °C until analyzed. Luminescence (luciferase activity) and absorbance (β-galactosidase activity) were detected with Hidex multiplate reader. For analysis, blank values were subtracted, and raw luciferase activities were normalized to absorbance to account for transfection efficiency and technical variability.

**Table 2:** NR expression vectors and luciferase reporters used in receptor activation assays.

NR	NR	Luciferase	Positive control
Mouse Pxr	75 ng CMX-GAL4-mPxr LBD [38]	75 ng UAS4-tk-luciferase^7^	RU487 10 μM Sigma Aldrich M8047
Mouse Car	75 ng CMX-GAL4-mCar LBD [38]	75 ng UAS4-tk-luciferase^7^	TCPOBOP 1 μM [38]
Mouse Pparα	75 ng CMX-GAL4-mPparα LBD^a^	25 ng UAS4-tk-luciferase^7^	WY14743 10 μM ChemSyn laboratories 98-794-12-22
Human PXR	75 ng CMX-GAL4-hPXR LBD [37]	75 ng UAS4-tk-luciferase [38]	SR12813 5 μM Cayman chemicals 18115
Human CAR	75 ng CMX-GAL4-hCAR LBD [37]	75 ng UAS4-tk-luciferase [38]	CITCO 1 μM Tocris 3783
Human PPARα	75 ng CMX-GAL4- PPARα LBD^b^	25 ng UAS4-tk-luciferase [38]	WY14743 10 μM ChemSyn laboratories 98-794-12-22
Human PPARγ	75 ng CMX-GAL4- PPARγ LBD^b^	50 ng UAS4-tk-luciferase [38]	Troglitazone 5 μM Cayman chemicals 71750
Human FXR	50 ng CMX-GAL4-hFXR LBD^c^	100 ng UAS4-tk-luciferase [38]	GW4074 10 μM Tocris 2473
Human LXRα	50 ng CMX-GAL4-hLXRα LBD^d^	75 ng UAS4-tk-luciferase [[38], [39]]	GW3975 10 μM Tocris 2474
Human GR	5 ng pSG5-hGR FL [[40], [41]]	75 ng pARE2-TATA-LUC [[40], [41]]	Dexamethasone 0.1 μM Sigma Aldrich D4902

a The mouse Pparα ligand binding domain (residues 170–468) was cloned in-house.

b A kind gift from Prof. Dr. Braeuning, German Federal Institute for Risk Assessment (BfR).

c The human FXR ligand binding domain (residues 222–472) [42] was cloned in-house.

d The human LXRα ligand binding domain (residues 206–447) was cloned in-house.

### 2.16 Statistical analysis

Statistical analyses and data visualization were done in GraphPad Prism v.10.0.2. and in R. Data analysis of the metabolomics and transcriptomics data are described in sections 2.9. and 2.10. Bodyweight gain was analyzed by two-way repeated measures ANOVA, followed by Dunnett’s multiple comparisons test. All other non-omics data were analyzed for normal distribution using the Shapiro–Wilk test, followed by one-way ANOVA and Dunnett’s multiple comparisons test for normally distributed data and non-parametric Kruskal–Wallis test followed by Dunn’s multiple comparisons test for non-normally distributed data. P-value <0.05 was considered statistically significant.

## 3 Results

### 3.1 DINP exposure decreased bodyweight gain and glucose tolerance in obese male mice

**Figure 1:**
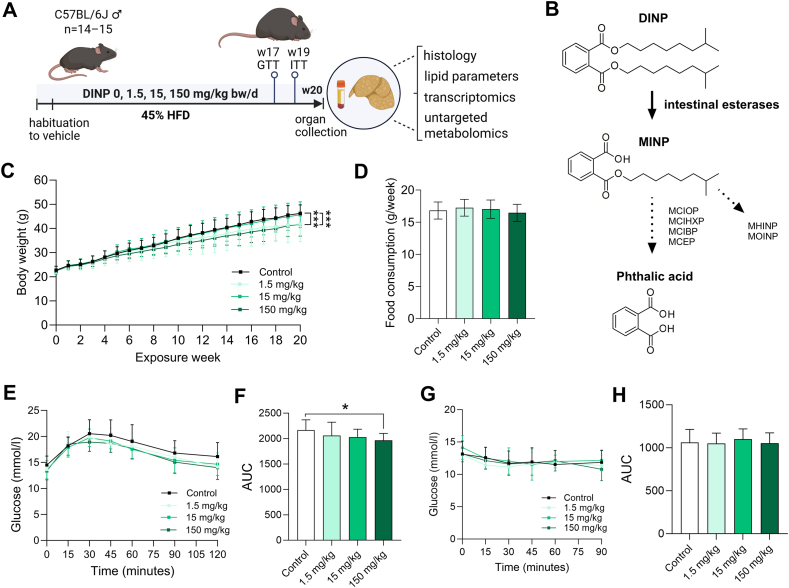
**20-week DINP exposure attenuates bodyweight gain and slightly improves glucose tolerance in obese male mice.** C57BL/6J male mice were exposed to 1.5, 15 or 150 mg/kg bw/d DINP for 20 weeks next to a high-fat diet to investigate DINP-induced metabolic effects in a DIO mouse model (**A**). Depiction of DINP metabolism, adapted from Saravanabhavan & Murray (2012), created in Biorender.com (Pitkänen, 2026) (**B**). Bodyweight gain was significantly lower in 1.5 and 150 mg/kg bw/d DINP groups during the 20-week study (**C**). Average food consumption per week (**D**). Intraperitoneal glucose tolerance test (GTT) with 1.5 g/kg glucose injection indicated slightly improved glucose tolerance in 150 mg/kg bw/d DINP group compared to the control (**E-F**). Intraperitoneal insulin tolerance test (ITT) with 0.5 mIU/g injection of rapid-acting insulin did not indicate differences in insulin tolerance between the groups (**G-H**). Data shown as mean ± SD, N = 14–15 mice per group. ∗ = p < 0.05, ∗∗ = p < 0.01, ∗∗∗ = p < 0.001. DINP – di-isononyl phthalate, MINP – monoisononyl phthalate.

Male C57BL/6J mice were exposed to 0, 1.5, 15 or 150 mg/kg bw/d DINP for 20 weeks next to HFD feeding (Figure 1A,B). Bodyweights of the mice gradually increased in all groups, but significantly lower bodyweight gain was observed in 1.5 and 150 mg/kg bw/d DINP groups (Figure 1C). The difference was not accompanied by significant differences in food intake (Figure 1D). During the 20-week exposure, the overall weight gain was +103.7 % in control, +84.2 % in 1.5 mg/kg bw/d DINP, +101.1 % in 15 mg/kg bw/d DINP and +83.7 % 150 mg/kg bw/d DINP groups. We compared the data to our historical data on the same mouse strain fed with regular chow diet [[23], [43]]. Our typical average weight gain during one week has been +0.5 g in chow-fed mice and +1.2 g in the current study with the high-fat diet control group. In the current study, the high-dose and low-dose DINP groups gained +0.95 g on average on a weekly basis, whereas the mid-dose group gained +1.1 g. This suggests that DINP exposure, particularly at the high and low doses, partially attenuated the diet-induced weight gain. DINP did not significantly affect any of the measured absolute or relative organ weights, including liver, heart, subcutaneous white adipose tissue (scWAT), gonadal WAT (gWAT) or brown adipose tissue (BAT) deposits (Table 3). However, the absolute mass of liver and BAT deposits tended to decrease especially in the highest dose group (−15 % and −22 %, compared to the control). During the study, we did not identify signs of systemic toxicity, or hepatotoxicity, since there were no changes in animal appearance or feeding behaviour, organ weights, or upregulation in expression of hepatic genes related to acute phase response or inflammation. Moreover, the levels of hepatotoxicity marker ALT in plasma were similar between the groups (Figure 3J). Glucose tolerance was slightly improved in the highest dose group, indicated by 9% decrease in area under curve (AUC) compared to the control in i.p.GTT (Figure 1E–F). Insulin tolerance was not significantly affected by DINP (Figure 1G–H). However, at 15 min, when the most drastic plasma glucose-lowering effect of rapid insulin is expected, a modest decrease in plasma glucose was seen in control group (−4.1 %) compared to all DINP-exposed groups (−12.1 %, −12.0 % and −11.8 %), indicating potentially faster glucose uptake in DINP-exposed groups compared to the control group (Figure 1G).

**Table 3:** Body and organ weights of C57BL/6J mice before and after 20-week DINP exposure and HFD feeding.

	Control	1.5 mg/kg DINP	15 mg/kg DINP	150 mg/kg DINP
Body weight, start (g)	22.70 ± 1.60	22.70 ± 1.30	22.71 ± 1.57	22.70 ± 1.28
Body weight, end (g)	46.25 ± 3.49	41.82 ± 7.27	45.66 ± 5.36	41.71 ± 4.84
Liver weight (g)	1.90 ± 0.51	1.70 ± 0.77	1.77 ± 0.54	1.61 ± 0.31
Relative liver weight (% bw)	4.07 ± 0.82	3.92 ± 1.12	3.80 ± 0.79	3.86 ± 0.46
Heart weight (g)	0.17 ± 0.02	0.16 ± 0.02	0.17 ± 0.02	0.16 ± 0.02
Relative heart weight (% bw)	0.38 ± 0.04	0.40 ± 0.08	0.37 ± 0.02	0.38 ± 0.02
scWAT weight (g)	2.49 ± 0.34	2.13 ± 0.54	2.37 ± 0.53	2.40 ± 0.54
Relative scWAT weight (% bw)	5.43 ± 0.97	5.17 ± 1.34	5.23 ± 1.29	5.76 ± 1.11
gWAT weight (g)	0.11 ± 0.04	0.09 ± 0.03	0.12 ± 0.06	0.11 ± 0.03
Relative gWAT weight (% bw)	0.24 ± 0.09	0.22 ± 0.09	0.26 ± 0.14	0.26 ± 0.09
BAT weight (g)	0.27 ± 0.07	0.22 ± 0.09	0.26 ± 0.08	0.21 ± 0.06
Relative BAT weight (% bw)	0.59 ± 0.14	0.50 ± 0.12	0.56 ± 0.12	0.50 ± 0.11

Data shown as mean ± SD, N = 14–15 mice per group. gWAT – gonadal white adipose tissue, scWAT – subcutaneous white adipose tissue, BAT – brown adipose tissue.

**Figure 2:**
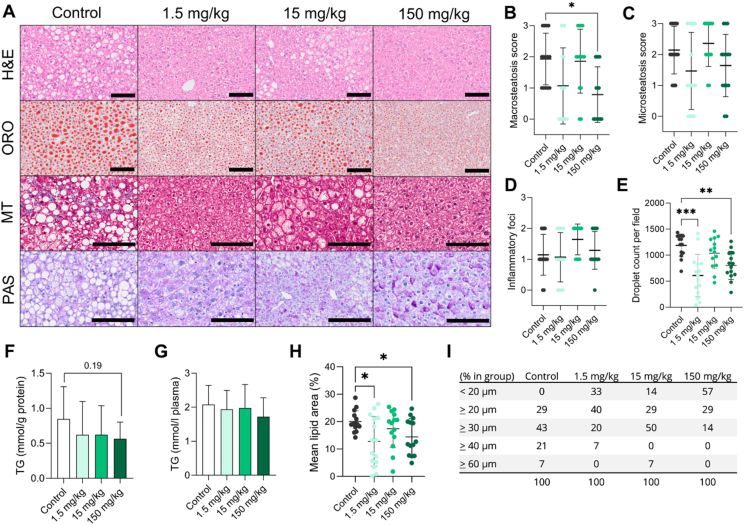
**Hepatic histology and lipid deposition are altered by DINP exposure**. Representative images of liver histology after hematoxylin-eosin (H&E), Oil-Red-O (ORO), Masson’s Trichrome (MT) and Periodic acid-Schiff (PAS) staining (H) (**A**). Visual scoring of H&E-stained liver sections indicated lower macrosteatosis grade in 150 mg/kg bw/d group compared to the control group (**B**). Visually scored intracellular microsteatosis (**C**) and inflammatory foci (**D**) were not altered between the groups. The number of fat droplets was lower in 1.5 mg/kg and 150 mg/kg DINP groups compared to the control (**E**). Liver and plasma triglycerides tended to gradually decrease by increasing doses of DINP (**F, G**). Measurement of the mean area of lipids indicated lower amount of steatosis in 1.5 and 150 mg/kg bw/d DINP groups compared to control (**H**). Diameter of the biggest lipid droplet was <20 μm in majority of the mice in 150 mg/kg bw/d DINP group, whereas all the control mice had lipid droplets at least 20 μM by diameter (**I**). Data shown as mean ± SD, N = 14–15 mice per group. ∗ = p < 0.05, ∗∗ = p < 0.01, ∗∗∗ = p < 0.001. Scale bar = 200 μm.

**Figure 3:**
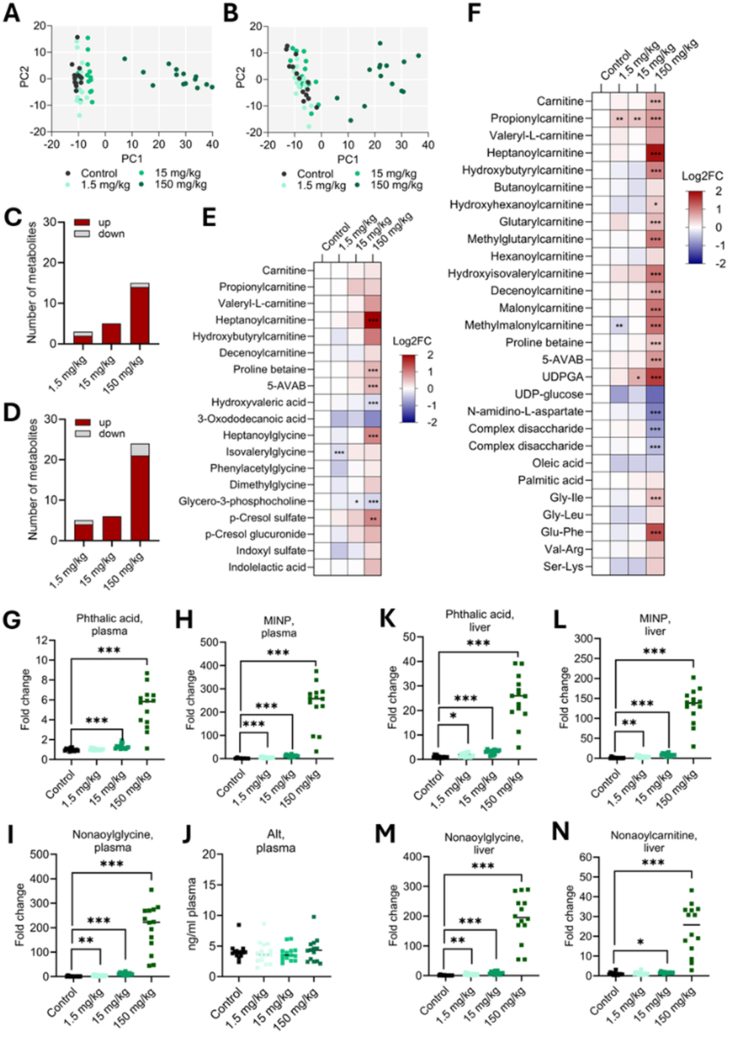
**DINP exposure induces changes in plasma and liver metabolome.** Principal component analysis (PCA) for top 500 features in plasma (**A**) and liver (**B**) indicated overall different metabolic profile mainly for 150 mg/kg bw/d DINP group compared to the control and lower dose groups. Number of significantly altered metabolites 1.5, 15 or 150 mg/kg bw/d DINP vs control group in plasma (**C**) and liver (**D**) (FDR <0.05). Heatmaps depict significant changes (FDR <0.05) and trends (P < 0.05) in the metabolite levels in plasma (**E**) and liver (**F**). The levels of known DINP metabolites phthalic acid and monoisononyl phthalate (MINP) were gradually increasing by DINP dose in the plasma (**G, H**) and in the liver (**K, L**). The level of nonaoylglycine was dose-dependently increased by DINP in the plasma (**I**) and the liver (**M**), as well as the level of nonaoylcarnitine in the liver (**N**). Marker of hepatotoxicity, alanine transaminase (Alt), was not altered between dose groups (**J**). Data shown as mean ± SD, N = 14–15 mice per group. ∗ = FDR <0.05, ∗∗ = FDR <0.01, ∗∗∗ = FDR <0.001.

### 3.2 DINP exposure affected hepatic histology and decreased lipid deposition

To analyze the effects of DINP on liver histology, H&E-stained sections were first inspected for general overview and analyzed for lipid deposition, by dly scoring the tissue for macro- and microsteatosis, and inflammatory foci (grade 0 to 3) (Figure 2A–D). Lipid droplet count, area of the lipid deposits, and diameter of the biggest droplets were quantified to assess liver adiposity more in detail (Figure 2E,H, I). The mice exposed to 150 mg/kg bw/d DINP had a lower macrosteatosis score (Figure 2B) and smaller mean area of the lipid deposits (Figure 2H) compared to the control group. The diameter of the biggest lipid droplet was <20 μm in 57 % of the mice in the same group, further indicating low amount of macrosteatosis and absence of large fat droplets in most of the subjects in this group (Figure 2I). In contrast, all mice in the control group had lipid droplets with at least 20 μm by diameter and 71 % of the mice in this group had liver lipid droplets with at least 30 μm by diameter, indicating the presence of marked macrosteatosis in all the subjects in this group (Figure 2I). Quantitative measurement of liver and plasma TGs showed a tendency for TG levels to decrease in both tissues gradually by increasing DINP dose (Figure 2F,G). MT staining highlighted minor collagen strains (shown in blue) mainly in the control and 15 mg/kg bw/d groups, whereas hardly any marks of collagen were seen in the highest dose group (Figure 2A). PAS staining indicated potential glycogen aggregates in 150 mg/kg bw/d group (shown as deep purple deposits) (Figure 2A).

### 3.3 DINP exposure affected metabolite profiles in mouse plasma and liver

20-week DINP-exposure led to significant increase in the level of multiple metabolites in plasma and liver (Figure 3). Principal component analysis (PCA) for top 500 molecular features detected in the untargeted metabolomics analysis indicated clear separation of the metabolite profiles in high-dose DINP group compared to the control and lower DINP dose groups in both plasma (Figure 3A) and liver (Figure 3B). Overall, we were able to annotate 15 metabolites in plasma and 24 metabolites in the liver, which were present at significantly different levels between at least one DINP-exposed group and the control group (FDR <0.05) (Figure 3C–F, Table S1). Additionally, we detected multiple (40+) metabolites, levels of which tended to be increased or decreased with p-value <0.05 but were not significantly altered after the FDR correction (>0.05) (Figure 3E–F, Table S1).

Out of the significantly affected metabolites, MINP, known as the primary active metabolite of DINP, increased by 3.5, 9.8 and 276-fold in the plasma and 3.6, 9.1 and 131-fold in the liver of the 1.5, 15 and 150 mg/kg bw/d DINP groups (Figure 3H,L). The gradually increasing relative abundance of MINP next to the increasing DINP exposure dose confirmed the phthalate exposure at three dose levels. Other strongly and gradually increased metabolites in all DINP exposure groups were phthalic acid, the final metabolite of all phthalates, which was increased up to 5.2-fold in the plasma and 24.8-fold in the liver (Figure 3G,K), nonaoylglycine, which was increased up to 200-fold in the plasma and 193-fold in the liver (Figure 3I,M), and nonaoylcarnitine, which was not detected in plasma but increased up to 24-fold in the liver (Figure 3N). The strong increase and structural resemblance imply that nonaoylglycine and nonaoylcarnitine may be conjugated DINP metabolites. DINP itself was not detected in the plasma or in the liver, which was expected due to the well-known rapid metabolism of diester phthalates in the gastrointestinal tract [44].

Carnitine has an important role in transporting long-chain fatty acids (LCFAs) in the form of acylcarnitines into mitochondria for energy production [45]. We observed significantly higher levels of several acylcarnitines in the 150 mg/kg bw/d DINP group compared to the control (Figure 3E–F). In plasma, the highest increase was observed in heptanoylcarnitine (6.8-fold), followed by hydroxybutyrylcarnitine (2.2-fold), valeryl-l-carnitine (1.7-fold) and propionylcarnitine (1.3-fold) with tendencies to increase also (Figure 3E). In the liver, the highest increase was observed in nonanoylcarnitine (24-fold), followed by heptanoylcarnitine (3.7-fold), methylmalonylcarnitine (2.2-fold), and hydroxyisovalerylcarnitine (2.1-fold), methylglutarylcarnitine (2.1-fold), malonylcarnitine (2.1-fold), hydroxybutyrylcarnitine (2.0-fold), propionylcarnitine (1.7-fold), decenoylcarnitine (1.6-fold), glutarylcarnitine (1.4-fold) and hydroxyhexanoylcarnitine (1.3-fold) (Figure 3F). The level of free carnitine was also 1.5-fold higher in the liver (Figure 3F).

In addition, a gut microbiota-derived compound 5-aminovaleric acid betaine (5-AVAB) has been coined as an emerging metabolite capable of regulating beta-oxidation by competing cellular uptake with carnitine [[46], [47]]. 5-AVAB was significantly higher in both plasma (1.5-fold) and liver (1.7-fold) in the high-dose DINP group (Figure 3E–F). Furthermore, glycerophosphocholine, which is known as acetylcholine precursor with potential capability to indirectly affect fatty acid oxidation [48], was reduced in the plasma of 15 and 150 mg/kg bw/d DINP groups and tended to decrease also in the liver.

In the liver, we also observed altered levels of several metabolites associated with conjugating metabolic reactions and detoxification (Figure 3F). Glucuronic acid donor uridine 5′-diphosphoglucuronic acid (UDPGA) was higher in the liver by 15 and 150 mg/kg bw/d DINP, which was accompanied by tendency in reduction in the level of its precursor uridine diphosphate glucose (UDP-glucose) in all DINP-exposed groups (Figure 3F). The level of glutathione, the key antioxidant molecule, tended to decrease in the liver, mainly in the mid- and high-dose DINP groups (Figure 3F).

The levels of two dipeptides including glycyl-isoleucine (Gly–Ile) and glutamylphenylalanine (Glu–Phe) were significantly higher, and several other dipeptides including glycyl-leucine (Gly–Leu), valine–arginine (Val–Arg) and serine–lysine (Ser–Lys) tended to increase in the livers of the highest dose group, indicating alterations also in amino acid metabolism (Figure 3F).

### 3.4 DINP exposure increased the expression of hepatic metabolic enzymes and transporters

**Figure 4:**
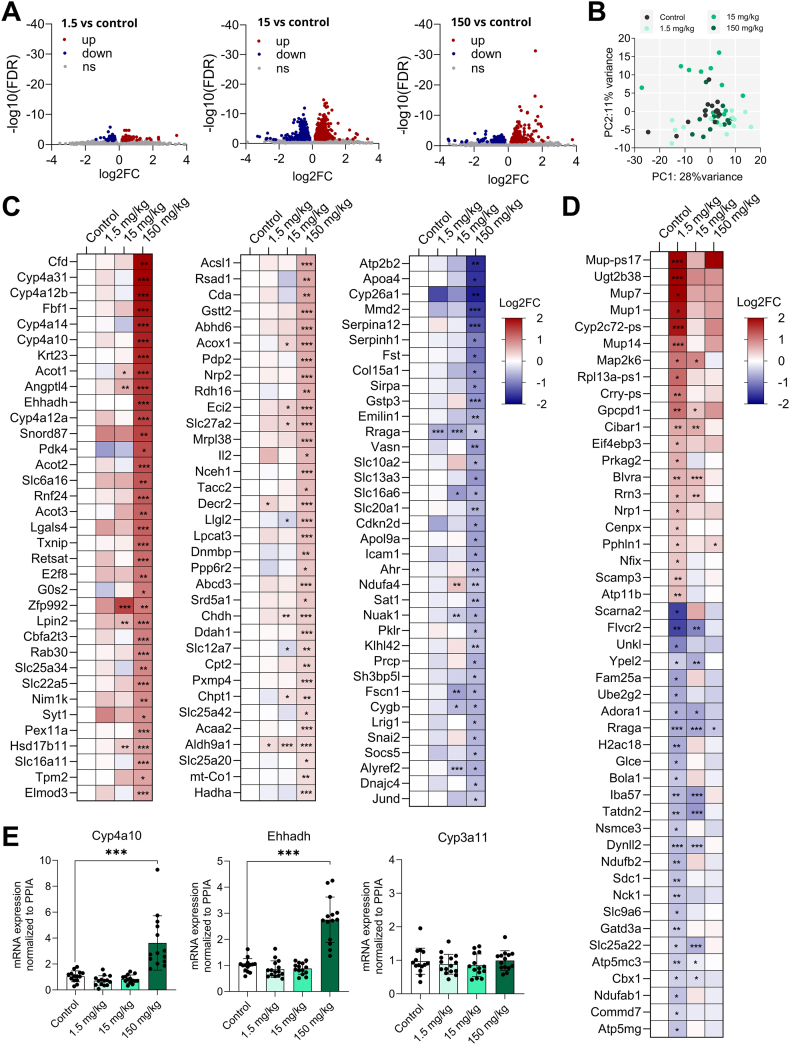
**DINP induces lipid metabolism-related changes in hepatic transcriptome.** Differentially expressed genes (FDR <0.05) between 1.5, 15 and 150 mg/kg bw/d DINP vs control group shown in volcano plots (**A**). Principal component analysis (PCA) indicates mild clustering between the groups (**B**). Heatmaps depicting top upregulated and downregulated genes in high-dose (**C**) and low-dose (**D**) groups (∗ = FDR <0.05, ∗∗ = FDR <0.01, ∗∗∗ = FDR <0.001). In line with the transcriptomics data, qPCR data verified the upregulation in Cyp4a10 and Ehhadh, and the absence of induction in Cyp3a11 (**E**) (∗∗∗ = p < 0.001).

The analysis for differentially expressed genes (DEGs) identified 36 upregulated and 30 downregulated genes in the 1.5 mg/kg bw/d group, 241 upregulated and 160 downregulated genes in the 15 mg/kg bw/d group, and 94 upregulated and 61 downregulated genes in the 150 mg/kg bw/d group (Figure 4A), when compared to the control group (FDR <0.05, log2FC >0.5). Principal component analysis (PCA) indicated mild clustering with partial overlap between the study groups (Figure 4B). The expression levels of key genes *Cyp4a10, Ehhadh* and *Cyp3a11* were confirmed by qPCR (Figure 4E).

Among the most significantly enriched genes by 150 mg/kg bw/d DINP was for example secreted complement factor D (*Cfd*), also known as adipsin, which is known for its ability to enhance insulin secretion [49] (Figure 4C). Also, several cytochrome P450 (CYP) family 4A members involved in microsomal lipid oxidation (*Cyp4a31, Cyp4a12b, Cyp4a14, Cyp4a10, Cyp4a12a*), mitochondrial lipid metabolizing enzymes enoyl-CoA delta isomerase 2 *(Eci2),* long-chain acyl-CoA synthetase (*Acsl1*), acetyl-CoA acyltransferase 2 (*Acaa2*), short-chain enoyl-CoA hydratase 1 *(Echs1)* and hydroxyacyl-CoA dehydrogenase trifunctional multienzyme complex subunit alpha (*Hadha*), peroxisomal enzymes enoyl-CoA hydratase (*Ehhadh*), enoyl-CoA delta isomerase 1 *(Eci1),* enoyl-CoA hydratase 1 *(Ech1),* acyl-CoA oxidase 1 *(Acox1)* and 2,4-dienoyl-CoA reductase *(Decr2),* and several members in acyl-CoA thioesterase family (*Acot1, Acot2, Acot3, Acot4, Acot12)* with associated functions in distinct cellular locations were among the top genes (Figure 4C). Other significantly enriched genes included for example angiopoietin-related protein 4 (*Angptl4*), which has a role as secreted inhibitor of lipoprotein lipase, and a local inhibitor of hepatic lipase (Kersten, 2021), steroid hormone metabolizing enzymes (*Hsd17b11*, *Hsdl2 and Srd5a1),* enzymes involved in triglyceride metabolism (*Lpin2, Pnpla2*) and phospholipid metabolism (*Lpcat3, Plpp3*), mitochondrial enzymes *(Chdh, Pdk4* and *Pdp2*) and cytochrome c oxidase (complex IV) subunit *(Mt–co1),* peroxisomal membrane proteins (*Pex11a, Abcd3 and Pxmp4*) and redox-regulating factors *(Txnip* and *Gstt2)* (Figure 4C).

**Figure 5:**
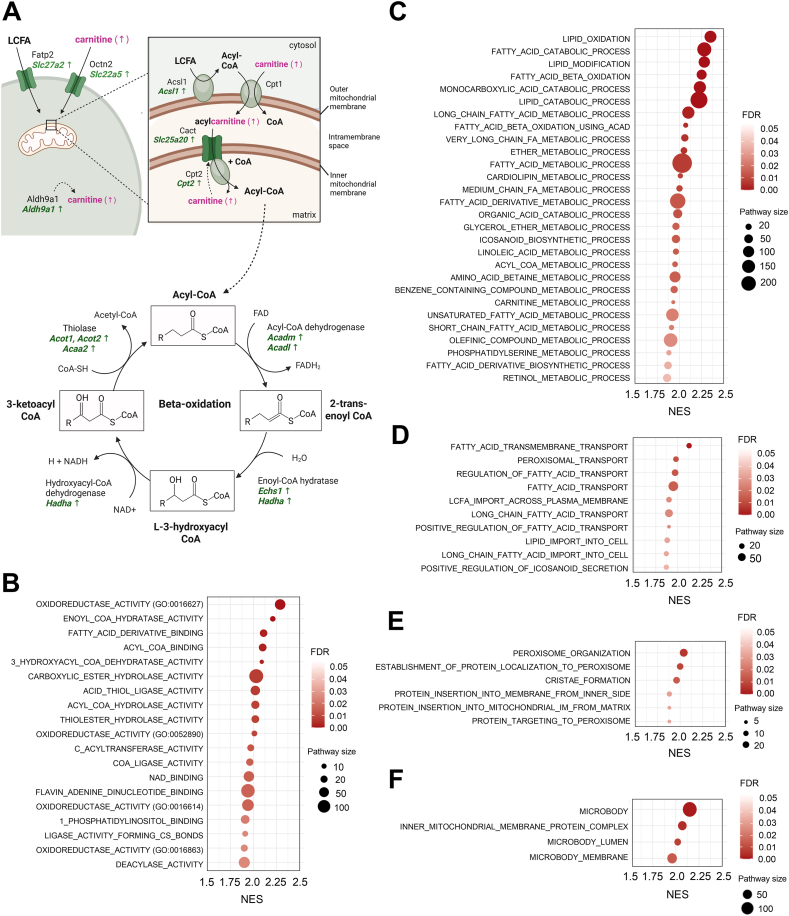
**DINP induces transcriptional changes in mitochondrial and peroxisomal lipid metabolism-related pathways.** DINP exposure affected multiple enzymes and transporters involved in carnitine shuttle and LCFA transport in transcriptomic and metabolomic levels (significantly enriched genes shown in green, metabolites in pink), depiction created in BioRender.com (Pitkänen, 2026) (**A**). Gene set enrichment analysis (GSEA) identified multiple enriched GO molecular functions (**B**), GO biological processes related to metabolism (**C**), transport (**D**) and organization (**E**), and GO cellular compartments (**F**) between 150 mg/kg bw/d DINP vs control group (**A**) (FDR <0.05). NES – normalized enrichment score.

In line with the high levels of acylcarnitines in metabolomics, carnitine shuttle and LCFA transport were affected also at transcriptomic level (Figure 4C, Figure 5A). The shuttle consists of a conversion step from acyl-CoA to acylcarnitine, which is catalyzed by carnitine palmitoyltransferase 1 (Cpt1), then acylcarnitine is transported inside the inner mitochondrial membrane via carnitine-acylcarnitine translocase (Cact, encoded by *Slc25a20*)*,* and reversely converted to acyl-CoA by carnitine palmitoyltransferase 2 (Cpt2). Other related transporters are organic cation/carnitine transporter 2 (Octn2, encoded by *Slc22a5*), which is responsible for carnitine uptake into the cells, and fatty acid binding proteins (Fatp1 and Fatp2, encoded by *Slc27a1 and Slc27a2),* which transport fatty acids into the cells. Most of these genes were significantly enriched in the 150 mg/kg bw/d DINP group (Figure 4C, Figure 5A). Also, the gene encoding the penultimate enzyme in carnitine biosynthesis (*Aldh9a1*) was mildly but consistently enriched in all dose groups (Figure 4C, Figure 5A).

In addition to the transporters involved in the carnitine shuttle, DINP also affected the expression of multiple other solute carrier (SLC) family members (Figure 4C,D). The alterations included upregulation in amino acid transporter *Slc6a16,* mitochondrial carriers *Slc25a34* and *Slc25a42,* monocarboxylate transporter *Slc16a11,* vitamin C transporter *Slc23a1,* and ion transporter *Slc12a7*, and downregulation in bile carrier *Slc10a2,* dicarboxylate transporter *Slc13a3,* monocarboxylate transporter *Slc16a6* and phosphate transporter *Slc20a1* in the high-dose DINP group (Figure 4C)*.* Of these SLCs, *Slc27a2* (up), and *Slc12a7 and Slc16a6* (down) were already affected by 15 mg/kg bw/d DINP (Figure 4C). Mitochondrial glutamate transporter Slc25a22 was downregulated in low- and mid-dose groups but not in the high-dose group (Figure 4D).

Among the top downregulated genes in the 150 mg/kg bw/d DINP group were for example several genes related to extracellular matrix organization (*Col15a1, Serpinh1, Emilin1, Icam1)* and cell adhesion *(Icam1, Nuak1, Fscn1)*. Among these genes were also several transporters and metabolic regulators, including for example plasma membrane calcium transporter *Atp2b2*, apolipoprotein *Apoa4*, retinoid acid metabolic enzyme *Cyp26a1*, progestin and adipoQ receptor family member *Mmd2*, secreted hepatokine follistatin (*Fst*), glutathione-S-transferase (*Gstp3*), aryl hydrocarbon receptor (*Ahr*), and mitochondrial complex IV (cytochrome c oxidase) subunit *Ndufa4*.

### 3.5 DINP exposure caused enrichment of hepatic lipid metabolism pathways

Next, GSEA was used to identify pathway enrichment focusing on affected Gene Ontology (GO) terms including biological processes (BPs), cellular compartments (CCs), and molecular functions (MFs), with the size of a gene set at least 5 genes. The analysis identified significant enrichment of 46 BPs, 4 CCs, and 19 MFs in 150 mg/kg bw/d group when compared to the control (FDR <0.05) (Figure 5B–F). For the 15 mg/kg bw/d dose group, only one enriched BP was identified after the FDR correction, which was the establishment of protein localization to telomere (GO:1904851). No significantly enriched GO terms were identified between the 1.5 mg/kg bw/d dose group and the control. The BPs enriched in the 150 mg/kg bw/d group were primarily related to mitochondrial lipid metabolism (e.g. GO:0034440, GO:0009062, GO:0030258, GO:0009437), peroxisomal lipid metabolism (e.g. GO:0034440, GO:0009062), and localization and transport of fatty acids (e.g. GO:0015908, GO:1902001, GO:2000193) (Figure 5C).

In line with the affected BPs, the four enriched CCs in the 150 mg/kg bw/d group included inner mitochondrial membrane protein complex (GO:0098800), microbody (GO:0042579), microbody lumen (GO:0031907), and microbody membrane (GO:0031903), which are typical intracellular locations for these enriched BPs (Figure 5F). The microbody refers to a small membrane-bound cellular organelle, typically peroxisome in animal cells.

Also, most of the affected MFs in the 150 mg/kg bw/d DINP group were related to lipid metabolism and aligned with different steps of beta-oxidation (fatty acid activation in the cytosol, followed by repeated cycle of dehydrogenation, hydration, oxidation and thiolysis in the mitochondrion/peroxisome) (Figure 5A–B). For example, CoA ligase activity (GO:0016405) and acyl-CoA binding (GO:0120227) are involved in the fatty acid activation step, oxidoreductase activity (GO:0016627, GO:0052890, GO:0016614 and GO:0016863) and flavin nucleotide binding (GO:0050660) are related to the dehydrogenation step, enoyl-CoA hydratase activity (GO:0004300) is related to hydration step, 3-hydroxyacyl-CoA dehydratase activity (GO:0018812), oxidoreductase activity and nicotinamide adenine dinucleotide (NAD) binding (GO:0051287) are related to the oxidation step, and thiolester hydrolase activity (GO:0016790), acyl-CoA hydrolase activity (GO:0016289), and c-acyltransferase activity (GO:0016408) are related to the thiolysis step (Figure 5A–B).

Structurally, DINP and its metabolites share a core chemical structure – benzene ring, which is attached to one or two carboxylate side groups via ester bond (Figure 1B). Several enriched MFs including monocarboxylic acid catabolic process (GO:0072329), monocarboxylic acid transport (GO:0015718), benzene containing compound metabolic processes (GO:0042537), and carboxylic ester hydrolase activity (GO:0052689) could be at least partially related to metabolism and trafficking of DINP and its metabolites, in addition to the normal lipid metabolism.

### 3.6 DINP metabolite MINP increases LCFA-dependent mitochondrial respiration

**Figure 6:**
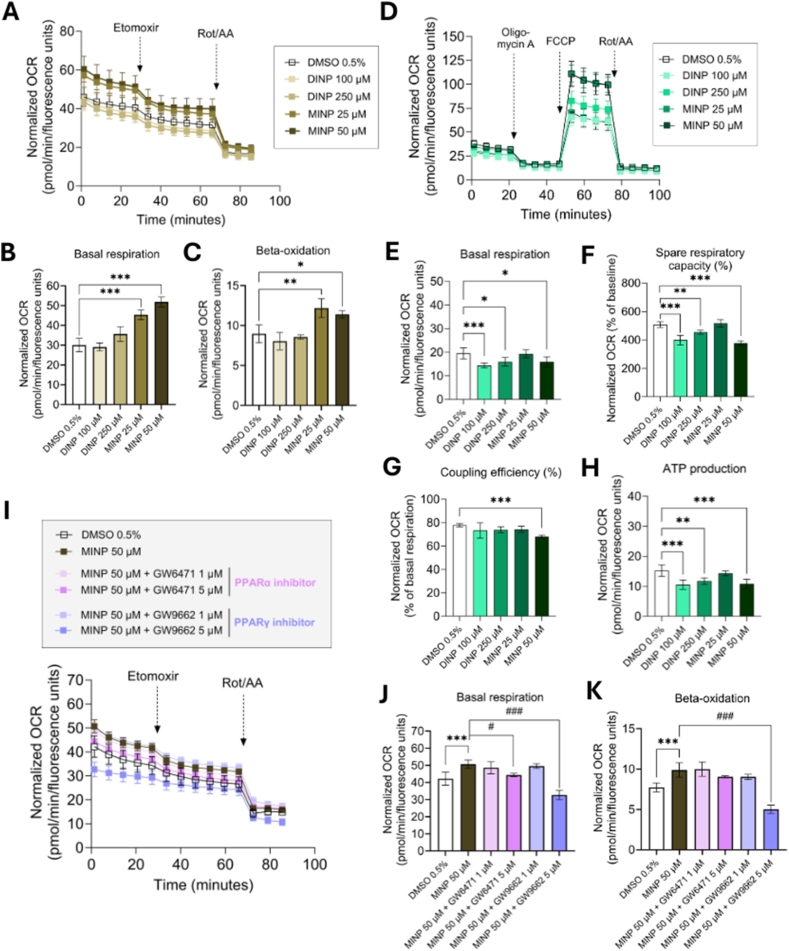
**DINP metabolite MINP increases LCFA-dependent mitochondrial respiration in human HepaRG cells.** In high-glucose conditions (**D-H**), DINP and MINP affected mitochondrial function by decreasing basal respiration (**E**), spare respiratory capacity (**F**), coupling efficiency (**G**) and ATP production (**H**), and in the presence of palmitate as energy substrate (**A-C, I-K**), MINP increased basal respiration (**E, J**) and beta-oxidation (**F, K**). MINP-induced increase in palmitate-driven respiration was abolished in the presence of PPARα inhibitor GW6471 and PPARγ inhibitor GW9662 (**J, K**). Data shown as mean ± SD, N = 4–5. ∗ = p < 0.05, ∗∗ = p < 0.01 and ∗∗∗ = p < 0.001 between phthalate-exposed cells and control. # = p < 0.05 and ### = p < 0.001 between MINP-exposed cells with and without the inhibitors. OCR – oxygen consumption rate.

Phthalate effects on mitochondrial function were then inspected by measuring mitochondrial respiration in HepaRG cells in the presence of different substrates for energy production (Figure 6). In the presence of relatively high glucose (10 mM), pyruvate (1 mM) and glutamine (2 mM), DINP and MINP decreased basal respiration (Figure 6E), spare respiratory capacity (Figure 6F), ATP production (Figure 6H) and coupling efficiency which reflects the proportion of respiration driving ATP synthesis (Figure 6G). In contrast, when the cells were depleted from glucose and pyruvate, and LCFA palmitate was used as a substrate instead (Figure 6A), the level of basal respiration and mitochondrial beta-oxidation significantly increased after MINP exposure (Figure 6B,C), reflecting similar effects that were seen in the mouse liver in transcriptomics pathway analysis (Figure 4). Finally, MINP-induced increase in LCFA-driven mitochondrial respiration was abolished in the presence of PPARα inhibitor GW6471 and PPARγ inhibitor GW9662 (Figure 6J,K), indicating their involvement in MINP-induced effects on mitochondrial function.

### 3.7 DINP metabolites MINP and MHINP activate human and mouse PPARs

**Figure 7:**
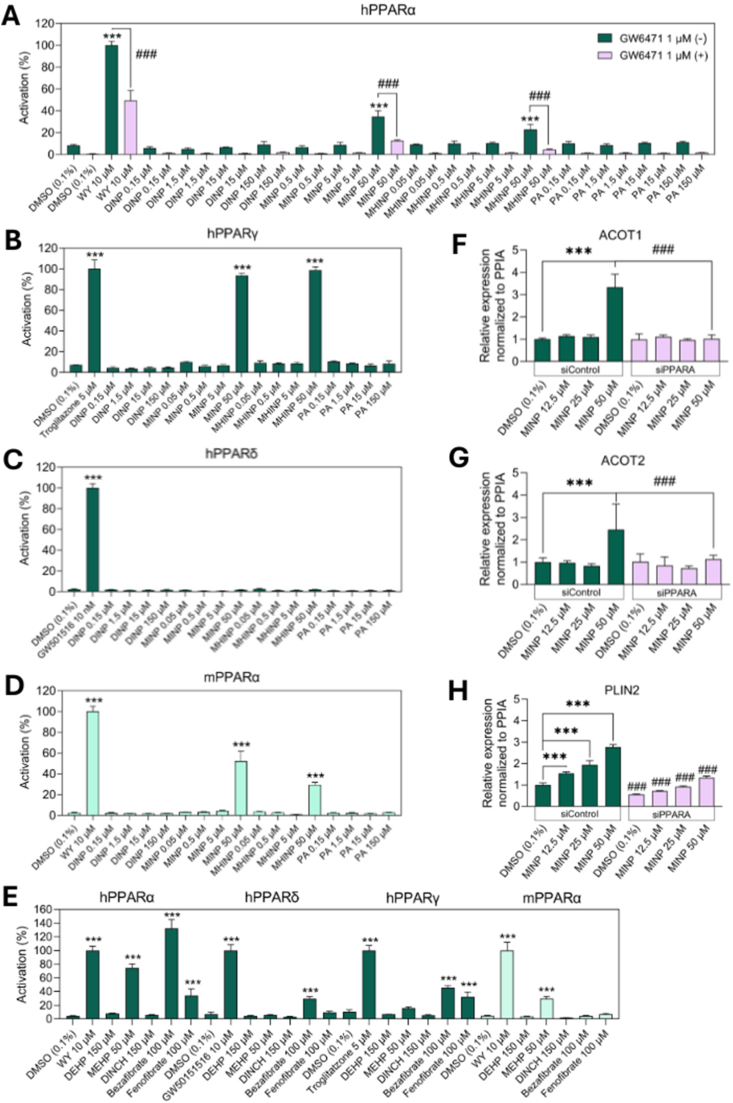
**DINP metabolites MINP and MHINP activate human and mouse PPARs in human C3A cells.** hPPARα is activated by MINP and MHINP but not DINP and PA, and the activation is reduced by hPPARα inhibitor GW6471 (**A**). MINP and MHINP also activate hPPARγ (**B**) and mPPARα (**D**) but not hPPARδ (**C**). The expression of PPARα target genes ACOT1, ACOT2 and PLIN2 is induced by MINP and reduced by PPARA knockdown (**F, G, H**). Similarly to MINP and MHINP, DEHP metabolite MEHP also activates hPPARα and mPPARα. PPARs are also targets of lipid-lowering drugs fibrates (**E**). Data shown as mean ± SD, N = 3. ∗ = p < 0.05, ∗∗ = p < 0.01, ∗∗∗ = p < 0.001. ### = p < 0.001 between the respective chemical treatments with and without PPARA knockdown. PPARα – peroxisome proliferator-activated receptor alpha, PPARγ – peroxisome proliferator-activated receptor gamma, PPARδ – peroxisome proliferator-activated receptor beta/delta.

Next, we inspected NR targets and DINP and its known metabolites MINP, MHINP and phthalic acid (PA) in human hepatic C3A cells. NRs are commonly activated or repressed by phthalates, among other MDCs [[7], [50]]. We observed that DINP metabolites MINP and MHINP strongly activated human PPARα and PPARγ, and mouse PPARα at 50 μM (Figure 7A–D). DINP itself did not activate any of the PPARs, and PA did not activate any NR. hPPARα activation was also tested in presence of inhibitor GW6471, which reduced the activation level induced by the metabolites (Figure 7A). MINP at 50 μM induced the expression of PPARα target genes ACOT1, ACOT2 and PLIN2, and the induction was significantly reduced by PPARα knockdown (Figure 7F–H).

We then compared the NR activation profile to other plasticizers, including DEHP, its primary metabolite MEHP, and phthalate-like 1,2-cyclohexane dicarboxylic acid diisononyl ester (DINCH), and clinical drugs used to treat dyslipidemias, including bezafibrate and fenofibrate (Figure 7E). First, we observed that both phthalates, DINP and DEHP, require metabolic conversion to activate PPARs. Furthermore, we observed more prominent activation of hPPARγ by the DINP metabolites MINP and MHINP, compared to the corresponding DEHP metabolite MEHP (Figure 7B,E). However, since DINP and DEHP metabolites were tested in different experiments, inter-experimental variability can decrease accuracy in comparisons of absolute potencies. The phthalate metabolites had similarities in their PPAR activation profile when compared to fibrates (Figure 7A–E).

**Figure 8:**
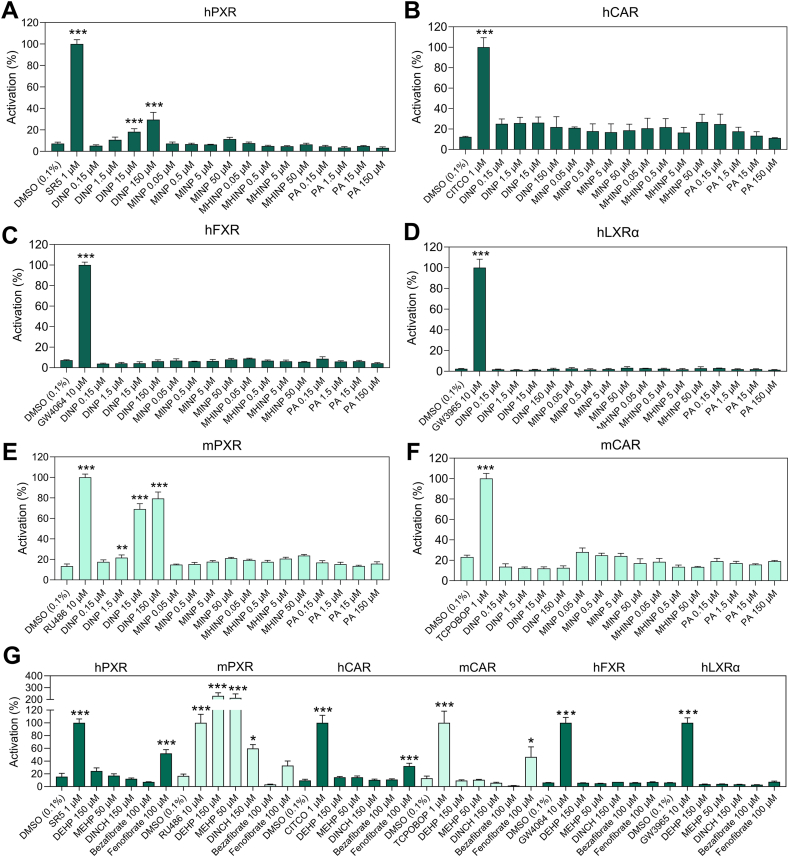
**Activation of human PXR, CAR, FXR and LXRα, and mouse PXR and CAR by phthalates and fibrates in human C3A cells.** DINP dose-dependently induced activation of hPXR (**A**) and mPXR (**E**), but no activity on hCAR (**B**), hFXR (**C**), hLXRα (**D**), and mCAR (**F**) was observed. DEHP and MEHP induced stronger activity of mPXR compared to DINP, whereas the reverse was observed in humans (**G**). Data shown as mean ± SD, N = 3. ∗ = p < 0.05, ∗∗ = p < 0.01, ∗∗∗ = p < 0.001. CAR – constitutive androstane receptor, FXR – farnesoid X receptor, LXRα – liver X receptor alpha, PXR – pregnane X receptor.

Next to PPARs, we also measured activation of human PXR, CAR, FXR and LXRα, and mouse PXR and CAR by phthalates and fibrates (Figure 8A–G). Out of these NRs, only human and mouse PXRs were activated by phthalates (Figure 8A,E). DINP activated both human and mouse PXR dose-dependently (Figure 8A,E). Importantly, we observed that DEHP and MEHP are stronger activators of mouse PXR compared to DINP, whereas the reverse was observed in humans (Figure 8A,E,G).

## 4 Discussion

By studying DINP using DIO mouse model and human hepatocyte cell lines, we observed notable effects on hepatic lipid metabolism. Collectively, this study demonstrated the capacity of DINP to (1) attenuate bodyweight gain, glucose tolerance and hepatic lipid deposition in mice, (2) remodel hepatic lipid metabolism and mitochondrial function in both mouse liver and human hepatic cells, and (3) activate NRs responsible for metabolic regulation. The functional observations from the metabolic tests were supported by the omics data from mouse liver and plasma, which revealed the enrichment of transcripts and metabolites associated with several lipid metabolism-related pathways. Overall, multiple pathways, as well as potentially saturating processes and compensatory mechanisms contribute to the overall DINP effect at different doses, resulting in a complex endocrine and metabolic interplay. The clearest and most consistent effect was the induction of hepatic lipid catabolism, which appears to be PPAR-driven, fully activated at a 150 mg/kg dose, with gradual signs present at lower doses. Central observation from the omics data was that especially the high dose affected mitochondrial and peroxisomal functions that are associated with metabolism of LCFAs. We identified several enzymes, transporters and metabolites, which were enriched in the transcriptomics and metabolomics analyses, and are known to be involved specifically in beta-oxidation of LCFAs and facilitate their transport through mitochondrial and peroxisomal membranes.

It has been demonstrated by previous studies that phthalates induce activation of different members of the PPAR family [[51], [52], [53]] and mediate metabolic effects through them in rodents [[19], [51]]. We observed that the gene expression level of the mouse PPARα increased by 49 %, and a high number of well-established PPAR-regulated genes (e.g. *Cyp4a* family, *Acot1, Acox1, Ehhadh, Angptl4, Txnip, Hadha)* [[54], [55], [56], [57]] were among the top enriched genes in our transcriptomics data. We also noticed that DINP metabolites MINP and MHINP strongly activated all tested mouse and human PPAR subtypes *in vitro*, whereas this effect was not consistent with the parental compound. At the same time, not all PPAR-mediated effects in the mouse liver are considered human relevant [58], and hepatic effects of PPAR activators are often milder or lacking in humanized rodent models [19]. Especially PPARα-mediated induction in peroxisomal proliferation, hepatomegaly and hepatocarcinogenesis are considered to be rodent-specific effects [58]. However, changes in lipid metabolism regulated by PPARα have been shown to be distinct from the proliferation response, and likely more human relevant [59].

In addition to the PPARs, we have identified several other factors likely contributing to the effect in the low-dose group. A first potential factor is the major urinary protein (MUP) family of pheromone carriers, which were upregulated exclusively in the low-dose group, with Mup1, Mup7, Mup11, Mup14, and Mup-ps17 induced between 2.3- and 10.6-fold. MUP upregulation has been shown to increase energy expenditure in obese mice [60,61], and MUP expression is regulated by androgens and testosterone [62], which are known targets of phthalate-induced endocrine disruption [63]. This suggests that low-dose DINP may promote energy expenditure through androgen-mediated MUP induction. A second potential contributor is Rraga, which was prominently downregulated in the low- and mid-dose groups. Rraga is a key component of the mTORC1 nutrient-sensing pathway, and its downregulation may promote a catabolic state and autophagy induction, potentially contributing to the metabolic phenotype observed at the low dose [64]. A third potential explanation is the saturation of hepatic metabolism of active DINP metabolites (MINP, MHINP, and others) at higher doses, which could lead to their accumulation and drive PPAR-mediated effects preferentially at the high dose. Several members of the UDP-glucuronosyltransferase (UGT) enzyme family responsible for glucuronidation and excretion of phthalate metabolites are upregulated in the low- and mid-dose groups, whereas only trends toward upregulation are observed in the high-dose group. This pattern is consistent with metabolic saturation at the highest dose, potentially shifting the balance toward metabolite accumulation.

The increase in mitochondrial respiration and beta-oxidation in the HepaRG cells after phthalate exposure reflected similar effects seen in the mouse liver, even though the respiratory capacity was not directly measured from the mouse. Importantly, the effect in cells was only detected in the presence of palmitate as the main substrate for energy production. This indicates that phthalates could have varying effects in the presence of different substrates and energetic states, even in the same organism. The observation is also consistent with previous animal studies with other suspected MDCs [65,66], highlighting diet as an important interacting factor affecting responses to MDCs.

Acylcarnitine levels have been previously demonstrated to be increased by perinatal exposures to DINP and DEHP [[67], [68]]. The increase is also consistent with our observations, and some pharmacological treatments activating PPARs in mice and rats [[69], [70]]. Clofibrate treatment has been shown to increase carnitine levels also in pig livers, which have closer resemblance to human with generally milder response to PPARα activators [71]. In contrast, also decreases in acylcarnitines have been reported after phthalate exposure in rodent livers but also in human cord blood [72]. These studies indicate that even short-term phthalate exposure during sensitive periods could have long-term metabolic effects. The carnitine enrichment effect in mouse has been proposed to be due to increased carnitine biosynthesis and hepatocellular uptake by OCTN2 [69]. Our transcriptomics data did not indicate consistent enrichment in enzymes involved in carnitine biosynthesis but showed more effect on the transporters on the cell membrane and mitochondria.

Previously in human cells, phthalates have been shown to be relatively potent activators of the xenobiotic sensors human CAR splice variant 2 (hCAR2) and pregnane X receptor (PXR) [73]. In our study, DINP did not affect CAR, but activated both human and mouse PXR *in vitro*. In the mouse liver, we could not confirm the induction in typical mouse PXR or CAR target genes, such as *Cyp3a11* and *Cyp2b10*. However, this could be explained by the rapid metabolism of DINP in the gastrointestinal system, and lack of activity of the metabolites on PXR and CAR. Another potential regulator for DINP effects could be third xenobiotic sensor, aryl hydrocarbon receptor (AHR), which has been shown to regulate hepatic mitochondria by controlling mitophagy via its direct target mitophagy receptor BNIP3 [74]. In our RNA-seq data, the level of *Ahr* was reduced, and *Bnip3* was slightly increased the high-dose DINP group.

Our receptor activation data suggests that there are significant differences between DINP- and DEHP-mediated responses in mouse and human. First, DEHP is a stronger activator of mouse PXR compared to DINP, whereas the reverse is observed in humans. This species-specific divergence is toxicologically significant, as PXR activation has been linked to lipogenic and hypercholesterolemic effects [75], suggesting that DEHP may drive stronger PXR-mediated metabolic disruption in mice, but in human this could be the opposite. Second, regarding PPAR activation, the key difference observed was a more prominent activation of human PPARγ by the DINP metabolites MINP and MHINP compared to DEHP metabolite MEHP. Given that PPARγ is a master regulator of adipogenesis, this finding suggests that DINP may carry higher adipogenic potential than DEHP specifically in humans, which is a difference that would not be apparent from rodent data alone. Taken together, these receptor-level differences indicate that DINP presents a distinct, and in some respects more concerning profile in the human context compared to DEHP, suggesting that DINP may not be toxicologically safer substitute for DEHP.

Our analyses also identified elevated levels of circulating factors (e.g., heptanoylcarnitine), as well as hepatic gene enrichment of factors that are typically secreted, e.g., adipsin (*Cfd*) and angiopoietin-like-4 (*Angptl4*). Previously, increase in adipsin has been shown to be linked with insulin resistance in the adipose tissue, which has been described to be its primary site of synthesis and action [76]. In our data, the baseline expression of the *Cfd* gene in the control group was modest (∼5 TPM), but in the high-dose DINP group it increased to the level of ∼25 TPM. The increase in expression of secreted mediators, as well as in plasma metabolites indicates that DINP effects are also exerted outside of the liver to the other target organs.

Previous DINP exposure studies using mice fed with standard low-fat diets have reported a variety of hepatic damage including enlargement of the liver, broadened hepatic cords and central veins, loose cytoplasm, and oedema [[77], [78]]. One study also reported moderate to severe infiltration of inflammatory cells and steatosis [79], of which the latter is also consistent effect with adult zebrafish [80]. These effects have been attributed to, for example, oxidative damage caused by lipid peroxidation and peroxisomal proliferation at the molecular/organelle level. In general, these histopathological observations were not replicated in our DIO model, in which DINP seemed to have effects that could be considered mostly beneficial (e.g. decrease in hepatic lipid droplets, protection from diet-induced obesity and enrichment in regulators of lipid metabolism). The stimulatory effects seen here are likely dependent on PPARα and the dietary status of the model, since similar effects have been demonstrated before in DEHP exposure studies [19,20], and this was the first study to investigate DINP effects in the DIO model. In line with the differential effects in rodent models, phthalate effects on mitochondrial function were also different in the presence of different substrates, reflecting similar patterns to what has been seen in the mouse models.

Collectively, this study demonstrated the capacity of DINP to remodel hepatic lipid metabolism and disrupt mitochondrial bioenergetic functions in both mouse liver and human hepatic cell lines. Our findings highlight that DINP metabolites specifically target PPARs at high doses and have additional modes of action at lower exposure levels, with species-specific differences in their PPAR isoform specificity. Taken together, this underscores the importance of comprehensive characterization of phthalates for their metabolic consequences, including determination of metabolite activities.

## CRediT authorship contribution statement

**Sini Pitkänen:** Writing – review & editing, Writing – original draft, Visualization, Methodology, Investigation, Formal analysis, Data curation, Conceptualization. **Henriikka Hakomäki:** Writing – review & editing, Supervision, Methodology, Investigation, Formal analysis, Data curation, Conceptualization. **Olli Kärkkäinen:** Writing – review & editing, Resources, Methodology. **Marko Lehtonen:** Writing – review & editing, Writing – original draft, Resources, Methodology, Data curation. **Sreejita Das:** Investigation, Data curation. **Jaana Rysä:** Writing – review & editing, Resources, Funding acquisition. **Jenni Küblbeck:** Writing – review & editing, Writing – original draft, Supervision, Resources, Methodology, Funding acquisition, Data curation. **Anna-Liisa Levonen:** Writing – review & editing, Supervision, Resources, Funding acquisition, Conceptualization.

## Funding sources

The project EDCMET has received funding from the European Union's Horizon 2020 research and innovation programme under Grant Agreement No. 825762. The project NEMESIS has received funding from the European Union's Horizon Europe programme under grant agreement No. 101137405. The computational analyses were performed on servers provided by UEF Bioinformatics Center, Biocenter Kuopio, Biocenter Finland, 10.13039/100007753University of Eastern Finland, Finland. We appreciate Biocentre Finland and Biocentre Kuopio for supporting the LC-MS laboratory facility.

## Declaration of competing interest

The authors declare the following financial interests/personal relationships which may be considered as potential competing interests: O.K. is a co-founder of Afekta Technologies Ltd., a company providing metabolomics analysis services.

If there are other authors, they declare that they have no known competing financial interests or personal relationships that could have appeared to influence the work reported in this paper.

## Data Availability

Data will be made available on request.
