# Supplementary material for: Metabolite-driven remodeling of hepatic lipid metabolism by the plasticizer di-isononyl phthalate
Source: Mol Metab. 2026 Jul 3;110:102412. doi: 10.1016/j.molmet.2026.102412 (PMC13382126; doi:10.1016/j.molmet.2026.102412)
Supplement: Multimedia component 2 [file mmc2.docx]

**Supplementary material 1**

**Metabolite-driven remodeling of hepatic lipid metabolism by the plasticizer di-isononyl phthalate**

Sini Pitkänen^1^, Henriikka Hakomäki^1^, Olli Kärkkäinen^2^, Marko Lehtonen^2^, Sreejita Das^1^, Jaana Rysä^2^, Jenni Küblbeck^1,2^, Anna-Liisa Levonen^1*^

Affiliations:

^1^A. I. Virtanen -institute for Molecular Sciences, University of Eastern Finland, Kuopio, Finland

^2^School of Pharmacy, University of Eastern Finland, Kuopio, Finland

^*^Corresponding author

Correspondence: [anna-liisa.levonen@uef.fi](mailto:anna-liisa.levonen@uef.fi)


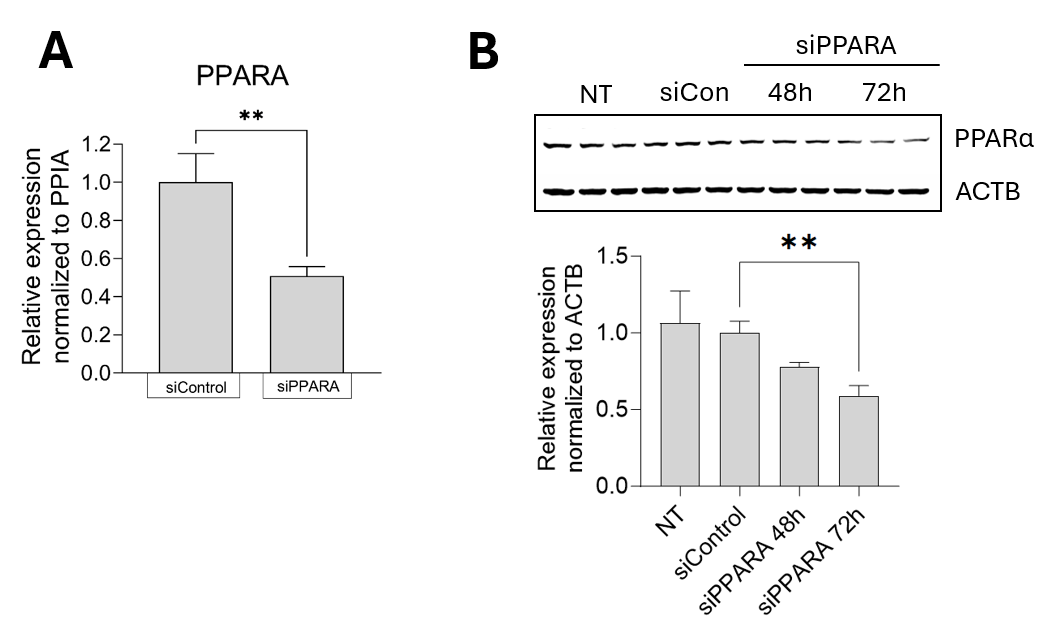


**Figure S1**. **Silencing efficiency of PPARα-targeting siRNA** **in C3A cells.** Human hepatic C3A cells were transfected with siRNA targeting PPARα or negative control (siCon) for 48 hours, followed by media change and incubation with 0.5% DMSO in treatment medium. The expression levels of PPARα were measured at 72 h for mRNA (**A**) and at 48 and 72 h for protein (**B**). The knockdown efficiency was 51 % for mRNA and 41 % for protein at 72 h. Data shown as mean ± SD of three replicates, ** = p < 0.01.
